# Supplementary material for: Mild anemia and 11- to 15-year mortality risk in young-old and old-old: Results from two population-based cohort studies
Source: PLoS One. 2021 Dec 31;16(12):e0261899. doi: 10.1371/journal.pone.0261899 (PMC8719676; doi:10.1371/journal.pone.0261899)
Supplement: S1 Fig — (DOCX) [file pone.0261899.s001.docx]

Eligible population *M80+*

alive at first interview

**N = 2,039**

Participants *H&A*

**N = 5,221**

Deceased, not found, and refusals **N = 4,871**

Eligible population *H&A*

**N = 10,092**

Refused laboratory tests

**N = 924**

Participants *M80+*

**N = 1,115**

*H&A 85+*

**N = 727**

*H&A 65-84*

**N = 4,494**

*Pooled cohort 80+*

**N = 1,842**

*M80+*

Participants

with two blood samples

**N = 366**

*H&A 65-84*

Participants

with two blood samples

**N = 692**

**S1 Fig. Flow chart of the *Health and Anemia* (*H&A*) and *Monzino 80-plus* (*M80+*) studies.**
