# Supplementary material for: Mild anemia and 11- to 15-year mortality risk in young-old and old-old: Results from two population-based cohort studies
Source: PLoS One. 2021 Dec 31;16(12):e0261899. doi: 10.1371/journal.pone.0261899 (PMC8719676; doi:10.1371/journal.pone.0261899)
Supplement: S1 Table — (DOCX) [file pone.0261899.s002.docx]

**S1 Table. Risk of mortality in anemic and mild anemic compared to non-anemic participants in pooled *Health & Anemia* and *Monzino-80 plus* population-based studies.**

|  |  | *Health & Anemia 65+* **and** *Monzino 80+* (N=6,113) | | | | |  |
| --- | --- | --- | --- | --- | --- | --- | --- |
| Anemia definitions | Model | 0-11 years | 0-7 years | | 8-11 years | |  |
| Anemia: [Hb] g/dL |  | Hazard ratios (95% confidence intervals) | | | | | |
| ≤11.9 (W) or ≤12.9 (M) ^a^ | ASA | 1.50 (1.37-1.64) | | 1.64 (1.48-1.81) | | 0.98 (0.78-1.25) | |
|  | F-A | 1.34 (1.22-1.47) | | 1.46 (1.32-1.62) | | 0.83 (0.64-1.07) | |
| ≤12.1 (W) or ≤13.1 (M)^b^ | ASA | 1.48 (1.35-1.61) | | 1.58 (1.44-1.74) | | 1.11 (0.91-1.36) | |
|  | F-A | 1.31 (1.19-1.43) | | 1.39 (1.25-1.54) | | 0.97 (0.78-1.21) | |
| Mild anemia: [Hb] g/dL |  | Hazard ratios (95% confidence intervals) | | | | | |
| 10.0^c^-11.9 (W) or 10.0-12.9 (M) | ASA | 1.44 (1.31-1.58) | | 1.56 (1.41-1.73) | | 0.96 (0.75-1.23) | |
|  | F-A | 1.29 (1.17-1.43) | | 1.40 (1.26-1.57) | | 0.79 (0.60-1.04) | |
| 11.0^d^-11.9 (W) or 11.0-12.9 (M) | ASA | 1.32 (1.19-1.47) | | 1.46 (1.30-1.63) | | 0.82 (0.61-1.09) | |
|  | F-A | 1.22 (1.09-1.36) | | 1.35 (1.19-1.52) | | 0.68 (0.50-0.93) | |
| 10.0^c^-12.1 (W) or 10.0-13.1 (M) | ASA | 1.42 (1.30-1.55) | | 1.51 (1.37-1.67) | | 1.10 (0.89-1.35) | |
|  | F-A | 1.26 (1.15-1.38) | | 1.33 (1.20-1.48) | | 0.95 (0.76-1.19) | |
| 11.0^d^-12.1 (W) or 11.0-13.1 (M) | ASA | 1.33 (1.20-1.46) | | 1.42 (1.27-1.58) | | 1.01 (0.80-1.27) | |
|  | F-A | 1.17 (1.08-1.33) | | 1.27 (1.14-1.43) | | 0.89 (0.69-1.13) | |

[Hb]: concentration of hemoglobin; W: women; M: men; AS-A: age- and sex-adjusted; F-A: "fully"-adjusted for baseline age, sex, education, smoking status, alcohol consumption, hypertension, diabetes, heart failure, myocardial infarction, chronic respiratory failure, chronic renal insufficiency, cancer, transient ischemic attack, stroke, parkinsonism, dementia, hospitalization during the previous year, and study.

^a^WHO criteria (1968) [22].

^b^Beutler and Waalen criteria (2006) for white adults [26].

^c^Dallman (1984); Groopman and Itri (1999); Wilson et al. (2004) [23-25].

^d^WHO criteria (2011) [27].
