# Supplementary material for: Mild anemia and 11- to 15-year mortality risk in young-old and old-old: Results from two population-based cohort studies
Source: PLoS One. 2021 Dec 31;16(12):e0261899. doi: 10.1371/journal.pone.0261899 (PMC8719676; doi:10.1371/journal.pone.0261899)
Supplement: S4 Table — (DOCX) [file pone.0261899.s005.docx]

**S4 Table. Risk of mortality in anemic and mild anemic compared with non-anemic participants aged 65-79 years at blood sample from the *Health and Anemia* population-based study and participants aged 80 years or older at blood sample from two pooled population-based studies (*Health and Anemia* *80+* and *Monzino 80-plus*).**

| **Anemia definitions** |  | ***Health & Anemia 65-79***  (N = 3,857) | | | ***Health & Anemia (80-84 and 85+)* and *Monzino 80+*** (N = 2,256) | | |
| --- | --- | --- | --- | --- | --- | --- | --- |
|  | Model | 0-15 years | 0-7 years | 8-15 years | 0-11 years | 0-7 years | 8-11 years |
| Anemia: [Hb] g/dL |  | Hazard ratios (95% confidence intervals) | | | Hazard ratios (95% confidence intervals) | | |
| ≤11.9 (W) or ≤12.9 (M)^a^ | AS-A | 1.76 (1.50-2.07) | 2.30 (1.83-2.89) | 1.41 (1.12-1.78) | 1.44 (1.30-1.59) | 1.56 (1.4-1.74) | 0.73 (0.51-1.04) |
|  | F-A | 1.44 (1.21-1.72) | 1.83 (1.42-2.36) | 1.22 (0.95-1.56) | 1.32 (1.18-1.47) | 1.43 (1.27-1.60) | 0.57 (0.38-0.85) |
| ≤12.1 (W) or ≤13.1 (M)^b^ | AS-A | 1.64 (1.42-1.90) | 2.09 (1.69-2.58) | 1.37 (1.12-1.67) | 1.41 (1.28-1.56) | 1.51 (1.36-1.68) | 0.88 (0.65-1.18) |
|  | F-A | 1.40 (1.20-1.64) | 1.73 (1.37-2.19) | 1.21 (0.98-1.50) | 1.26 (1.13-1.39) | 1.34 (1.20-1.50) | 0.74 (0.53-1.02) |
| Mild anemia: [Hb] g/dL |  | Hazard ratios (95% confidence intervals) | | | Hazard ratios (95% confidence intervals) | | |
| 10.0^c^-11.9 (W) or 10.0-12.9 (M) | AS-A | 1.64 (1.38-1.94) | 2.08 (1.63-2.65) | 1.35 (1.06-1.72) | 1.39 (1.25-1.55) | 1.51 (1.35-1.69) | 0.68 (0.47-1.00) |
|  | F-A | 1.36 (1.13-1.63) | 1.67 (1.28-2.19) | 1.17 (0.91-1.52) | 1.28 (1.14-1.43) | 1.39 (1.23-1.56) | 0.52 (0.34-0.80) |
| 11.0^d^-11.9 (W) or 11.0-12.9 (M) | AS-A | 1.56 (1.29-1.87) | 2.02 (1.56-2.63) | 1.25 (0.96-1.62) | 1.29 (1.14-1.45) | 1.41 (1.24-1.60) | 0.58 (0.38-0.91) |
|  | F-A | 1.30 (1.06-1.59) | 1.64 (1.22-2.19) | 1.10 (0.83-1.46) | 1.23 (1.08-1.39) | 1.33 (1.17-1.52) | 0.48 (0.29-0.79) |
| 10.0^c^-12.1 (W) or 10.0-13.1 (M) | AS-A | 1.55 (1.33-1.80) | 1.91 (1.53-2.39) | 1.32 (1.07-1.63) | 1.37 (1.24-1.52) | 1.47 (1.31-1.63) | 0.85 (0.63-1.16) |
|  | F-A | 1.33 (1.13-1.57) | 1.61 (1.26-2.05) | 1.18 (0.94-1.47) | 1.22 (1.09-1.36) | 1.30 (1.16-1.46) | 0.71 (0.51-1.00) |
| 11.0^d^-12.1 (W) or 11.0-13.1 (M) | AS-A | 1.48 (1.26-1.74) | 1.85 (1.46-2.35) | 1.25 (1.00-1.56) | 1.28 (1.15-1.43) | 1.37 (1.22-1.54) | 0.80 (0.58-1.12) |
|  | F-A | 1.29 (1.08-1.54) | 1.57 (1.21-2.04) | 1.13 (0.89-1.43) | 1.17 (1.04-1.31) | 1.24 (1.10-1.41) | 0.73 (0.50-1.05) |

[Hb]: concentration of hemoglobin; W: women; M: men; AS-A: age- and sex-adjusted; F-A: "fully"-adjusted for baseline age, sex, education, smoking status, alcohol consumption, hypertension, diabetes, heart failure, myocardial infarction, chronic respiratory failure, chronic renal insufficiency, cancer, transient ischemic attack*,* stroke, parkinsonism, dementia, hospitalization during the previous year, and study (only for the two pooled studies).

^a^WHO criteria (1968) [22].

^b^Beutler and Waalen criteria (2006) for white adults [26].

^c^Dallman (1984); Groopman and Itri (1999); Wilson et al. (2004) [23-25].

^d^WHO criteria (2011) [27].
