# Supplementary material for: Mild anemia and 11- to 15-year mortality risk in young-old and old-old: Results from two population-based cohort studies
Source: PLoS One. 2021 Dec 31;16(12):e0261899. doi: 10.1371/journal.pone.0261899 (PMC8719676; doi:10.1371/journal.pone.0261899)
Supplement: S6 Table — (DOCX) [file pone.0261899.s007.docx]

**S6 Table. Risk of mortality over 15 and 11 years in anemic and mild anemic compared with non-anemic participants aged 80 years or older at blood sample from the *Monzino 80-plus* population based study.**

| **Anemia definitions** |  | ***Monzino 80+*** (N = 1,115) | | | ***Monzino 80+*** (N = 1,115) | | |
| --- | --- | --- | --- | --- | --- | --- | --- |
|  | Model | 0-15 years | 0-7 years | 8-15 years | 0-11 years | 0-7 years | 8-11 years |
| Anemia: [Hb] g/dL |  | Hazard ratios (95% confidence intervals) | | | Hazard ratios (95% confidence intervals) | | |
| ≤11.9 (W) or ≤12.9 (M)^a^ | AS-A | 1.51 (1.32-1.71) | 1.59 (1.39-1.83) | 0.99 (0.65-1.49) | 1.50 (1.31-1.71) | 1.59 (1.39-1.83) | 0.79 (0.47-1.31) |
|  | F-A | 1.39 (1.21-1.59) | 1.46 (1.26-1.69) | 0.87 (0.54-1.41) | 1.38 (1.20-1.58) | 1.46 (1.26-1.69) | 0.70 (0.38-1.26) |
| ≤12.1 (W) or ≤13.1 (M)^b^ | AS-A | 1.47 (1.29-1.66) | 1.53 (1.34-1.75) | 1.12 (0.77-1.61) | 1.46 (1.29-1.66) | 1.53 (1.34-1.75) | 0.98 (0.64-1.50) |
|  | F-A | 1.30 (1.14-1.48) | 1.35 (1.17-1.56) | 0.95 (0.62-1.44) | 1.30 (1.13-1.48) | 1.35 (1.17-1.56) | 0.86 (0.53-1.41) |
| Mild anemia: [Hb] g/dL |  | Hazard ratios (95% confidence intervals) | | | Hazard ratios (95% confidence intervals) | | |
| 10.0^c^-11.9 (W) or 10.0-12.9 (M) | AS-A | 1.47 (1.29-1.69) | 1.56 (1.35-1.80) | 0.95 (0.60-1.49) | 1.48 (1.29-1.70) | 1.56 (1.35-1.80) | 0.79 (0.45-1.38) |
|  | F-A | 1.36 (1.18-1.57) | 1.43 (1.23-1.66) | 0.87 (0.53-1.45) | 1.36 (1.18-1.57) | 1.43 (1.23-1.66) | 0.72 (0.38-1.36) |
| 11.0^d^-11.9 (W) or 11.0-12.9 (M) | AS-A | 1.34 (1.15-1.56) | 1.43 (1.22-1.68) | 0.85 (0.52-1.38) | 1.34 (1.15-1.57) | 1.43 (1.22-1.68) | 0.64 (0.33-1.23) |
|  | F-A | 1.27 (1.08-1.49) | 1.33 (1.12-1.57) | 0.85 (0.49-1.47) | 1.26 (1.07-1.48) | 1.33 (1.12-1.57) | 0.66 (0.32-1.36) |
| 10.0^c^-12.1 (W) or 10.0-13.1 (M) | AS-A | 1.43 (1.26-1.64) | 1.50 (1.30-1.72) | 1.10 (0.75-1.61) | 1.44 (1.26-1.64) | 1.50 (1.30-1.72) | 1.01 (0.64-1.58) |
|  | F-A | 1.27 (1.11-1.46) | 1.32 (1.14-1.53) | 0.96 (0.62-1.47) | 1.27 (1.11-1.46) | 1.32 (1.14-1.53) | 0.90 (0.54-1.50) |
| 11.0^d^-12.1 (W) or 11.0-13.1 (M) | AS-A | 1.32 (1.14-1.52) | 1.38 (1.18-1.60) | 1.03 (0.69-1.55) | 1.32 (1.14-1.52) | 1.38 (1.18-1.60) | 0.92 (0.57-1.49) |
|  | F-A | 1.18 (1.02-1.37) | 1.21 (1.03-1.43) | 0.94 (0.60-1.47) | 1.18 (1.01-1.37) | 1.21 (1.03-1.43) | 0.88 (0.51-1.49) |

[Hb]: concentration of hemoglobin; W: women; M: men; AS-A: baseline age- and sex-adjusted; F-A: "fully"-adjusted for baseline age, sex, education, smoking status, alcohol consumption, hypertension, diabetes, heart failure, myocardial infarction, chronic respiratory failure, chronic renal insufficiency, cancer, transient ischemic attack*,* stroke, parkinsonism, dementia, hospitalization during the previous year, and study (only for the two pooled studies).

^a^WHO criteria (1968) [22].

^b^Beutler and Waalen criteria (2006) for white adults [26].

^c^Dallman (1984); Groopman and Itri (1999); Wilson et al. (2004) [23-25].

^d^WHO criteria (2011) [27].
