# Supplementary material for: Mild anemia and 11- to 15-year mortality risk in young-old and old-old: Results from two population-based cohort studies
Source: PLoS One. 2021 Dec 31;16(12):e0261899. doi: 10.1371/journal.pone.0261899 (PMC8719676; doi:10.1371/journal.pone.0261899)
Supplement: S7 Table — (DOCX) [file pone.0261899.s008.docx]

**S7 Table. Risk of mortality in anemic and mild anemic compared with non-anemic participants aged 65-84 years at blood sample from the *Health and Anemia* population-based study and participants aged 80 years or older at blood sample from two pooled population-based studies (*Health and Anemia 85+* and *Monzino 80-plus*).**

|  |  | *Health & Anemia 65-84* (N = 4,144) | | | *Health & Anemia 85*+ and *Monzino 80+*^f^ | | |
| --- | --- | --- | --- | --- | --- | --- | --- |
| Anemia definitions |  | 0-15 years | 0-7 years | 8-15 years | 0-11 years | 0-7 years | 8-11 years |
| Anemia: [Hb] g/dL | F-A  Model | Hazard ratios (95% confidence intervals) | | | Hazard ratios (95% confidence intervals) | | |
| ≤11.9 (W) or ≤12.9 (M)^a^ | +BMI | 1.43 (1.24-1.66) | 1.74 (1.42-2.14) | 1.20 (0.97-1.50) | 1.32 (1.17-1.49) | 1.41 (1.24-1.61) | 0.69 (0.45-1.06) |
|  | ↔MDRD^e^ | na | na | na | 1.34 (1.19-1.50) | 1.43(1.27-1.63) | 0.64 (0.41-1.00) |
| ≤12.1 (W) or ≤13.1 (M)^b^ | +BMI | 1.34 (1.17-1.53) | 1.62 (1.34-1.95) | 1.14 (0.94-1.37) | 1.27 (1.13-1.43) | 1.34 (1.18-1.51) | 0.89 (0.63-1.26) |
|  | ↔MDRD^e^ | na | na | na | 1.29 (1.15-1.45) | 1.36 (1.21-1.54) | 0.83 (0.58-1.19) |
| Mild anemia: [Hb] g/dL |  | Hazard ratios (95% confidence intervals) | | | Hazard ratios (95% confidence intervals) | | |
| 10.0^c^-11.9 (W) or 10.0-12.9 (M) | +BMI | 1.35 (1.16-1.59) | 1.63 (1.31-2.03) | 1.16 (0.92-1.46) | 1.26 (1.11-1.43) | 1.35 (1.18-1.54) | 0.62 (0.39-0.98) |
|  | ↔MDRD^e^ | na | na | na | 1.29 (1.14-1.47) | 1.39 (1.22-1.59) | 0.57 (0.35-0.93) |
| 11.0^d^-11.9 (W) or 11.0-12.9 (M) | +BMI | 1.31 (1.11-1.56) | 1.61 (1.28-2.04) | 1.08 (0.84-1.39) | 1.23 (1.07-1.41) | 1.33 (1.15-1.54) | 0.60 (0.36-1.01) |
|  | ↔MDRD^e^ | na | na | na | 1.22 (1.07-1.41) | 1.31 (1.14-1.52) | 0.55 (0.32-0.94) |
| 10.0^c^-12.1 (W) or 10.0-13.1 (M) | +BMI | 1.31 (1.15-1.51) | 1.56 (1.28-1.90) | 1.13 (0.94-1.39) | 1.22 (1.08-1.37) | 1.28 (1.12-1.45) | 0.84 (0.58-1.21) |
|  | ↔MDRD^e^ | na | na | na | 1.25 (1.11-1.41) | 1.32 (1.16-1.50) | 0.79 (0.54-1.16) |
| 11.0^d^-12.1 (W) or 11.0-13.1 (M) | +BMI | 1.27 (1.11-1.48) | 1.54 (1.25-1.90) | 1.09 (0.88-1.34) | 1.18 (1.04-1.35) | 1.24 (1.08-1.43) | 0.87 (0.59-1.28) |
|  | ↔MDRD^e^ | na | na | na | 1.19 (1.05-1.35) | 1.25 (1.09-1.43) | 0.81 (0.54-1.21) |

[Hb]: concentration of hemoglobin; W: women; M: men; BMI: Body mass index; MDRD: Modification of Diet in Renal Disease formula; na: MDRD not available for *H&A 65-84*; AS-A: age- and sex-adjusted; F-A: "fully"-adjusted for baseline age, sex, education, smoking status, alcohol consumption, hypertension, diabetes, heart failure, myocardial infarction, chronic respiratory failure, chronic renal insufficiency, cancer, transient ischemic attack, stroke, parkinsonism, dementia, hospitalization during the previous year, and study (only for the two pooled studies).

^a^WHO criteria (1968) [22].

^b^Beutler and Waalen criteria (2006) for white adults [26].

^c^Dallman (1984); Groopman and Itri (1999); Wilson et al. (2004) [23-25].

^d^WHO criteria (2011) [27].

^e^Fully adjusted model with MDRD (as a continuous variable) in place of chronic renal insufficiency.

**^f^**Plus BMI: N=1,545; with MDRD: N=1,571.

**S References for S1-S7 Tables** (numbering as in the manuscript)

22. World Health Organization. Nutritional anaemias. Report of a WHO Scientific Group. *World Health Organ Tech Rep Ser*. 1968;405:5-37. PMID: 4975372

23. Dallman PR, Yip R, Johnson C. Prevalence and causes of anemia in the United States, 1976 to 1980. *Am J Clin Nutr*. 1984,39(3):437-445. doi: 10.1093/ajcn/39.3.437

24. Groopman JE, Itri LM. Chemotherapy-induced anemia in adults: incidence and treatment. *J Natl Cancer Inst*. 1999;91(19):1616-1634. doi: 10.1093/jnci/91.19.1616

25. Wilson A, Yu H-T, Goodnough LT, Nissenson AR. Prevalence and outcomes of anemia in rheumatoid arthritis: a systematic review of the literature. *Am J Med*. 2004;116(suppl 7A):50S-57S. doi: 10.1016/j.amjmed.2003.12.012

26. Beutler E, Waalen J. The definition of anemia: what is the lower limit of normal of the blood hemoglobin concentration? *Blood*. 2006;107(5):1747-1750. doi: 10.1182/blood-2005-07-3046

27. WHO. Haemoglobin concentrations for the diagnosis of anaemia and assessment of severity. Vitamin and Mineral Nutrition Information System. Geneva, World Health Organization, 2011 (WHO/NMH/NHD/MNM/11.1).
