# Supplementary material for: Mild anemia and 11- to 15-year mortality risk in young-old and old-old: Results from two population-based cohort studies
Source: PLoS One. 2021 Dec 31;16(12):e0261899. doi: 10.1371/journal.pone.0261899 (PMC8719676; doi:10.1371/journal.pone.0261899)
Supplement: S1 Methods — (DOCX) [file pone.0261899.s009.docx]

**Supplementary methods**

*Definitions of anemia types*

Iron deficiency anemia was considered present if participant had low serum iron (<50 μg/dL in women and <60 μg/dL in men), low ferritin (<15 ng/mL), low transferrin saturation rate (<16%) or increased total iron binding capacity (>450 μg/dL). Anemia of chronic disease was defined as low circulating iron in the presence of increased iron stores (normal or increased ferritin >100 ng/mL, transferrin saturation >25% and <50%) and decreased total iron binding capacity (<250 μg/dL). Thalassemia trait was considered when the following conditions were present: low or very low mean corpuscular volume (MCV) and mean corpuscular hemoglobin, increased red blood cell count, normal or increased circulating iron in the presence of normal or increased iron stores. Anemia associated with folate or vitamin B_12_ deficiency was defined as concentrations of folate <3.0 ng/mL or vitamin B_12_ <200 pg/mL and MCV >95 fL. Subjects were classified as having anemia of chronic kidney disease when affected by chronic renal insufficiency. The classification of anemia types based on the hematologic findings was supported by the clinical conditions and pharmacological therapies of the elderly. Anemias that could not be classified into any of the previous categories were considered to be of unexplained origin.

Based on these shared definitions, three study experts classified the different types of anemia for all cases independent of the judgment of the others. Subsequently, the physicians reviewed, discussed and reached a final consensus for each preliminary discrepant classification [Tettamanti M, Lucca U, Gandini F, Recchia A, Mosconi P, Apolone G, et al. Prevalence, incidence and types of mild anemia in the elderly: the “Health and Anemia” population-based study. Haematologica. 2010; 95(11):1849-56].

*Index of comorbidity*

An index of comorbid disease severity was developed for the purposes of the *H&A* study: based on the medical information collected by the nurses, co-morbid disease severity was graded by two physicians using a cumulative score with a 5-point scale. The definitions of rating points (from 1, no impairment, to 5, extremely severe) are very similar to those of the Cumulative Illness Rating Scale [Parmelee PA, Thuras PD, Katz IR, Lawton MP. Validation of the Cumulative Illness Rating Scale in a geriatric residential population. J Am Geriatr Soc. 1995;43(2):130-137].
