# Supplementary material for: Mild anemia and 11- to 15-year mortality risk in young-old and old-old: Results from two population-based cohort studies
Source: PLoS One. 2021 Dec 31;16(12):e0261899. doi: 10.1371/journal.pone.0261899 (PMC8719676; doi:10.1371/journal.pone.0261899)
Supplement: Attachment — Submitted filename: Response to Reviewer.pdf [file pone.0261899.s011.pdf]

Response to Reviewers

Reviewer #2: Thank you for the detailed responses to the previous comments. I have a few additional comments:

1. It would be useful to clarify the time-varying analysis. The text mentions that "limited to the Monzino 80plus study, beyond baseline, other 8 follow-up assessments were available. It was thus possible to [further] adjust the multivariable model also for time-varying covariates […] in subjects with one (prevalent cases) as well as change in hemoglobin concentration (incident cases and hemoglobin decline)." **[1.1]** It is unclear whether exposure was assessed at each of the 8 follow-up assessments, or "in subjects with one" (assessment? hemoglobin determination?). The text also adds "… in subjects with two hemoglobin determinations it was also possible to investigate how the association between baseline anemia and mortality was affected by time-varying covariates together with change in anemia/non-anemia status at second sampling." **[1.2]** Was the exposure treated as time-varying in the analysis to permit the use of time-varying covariates? Later in the text, it is mentioned that "in all subjects with two hemoglobin determinations it was possible to control for both changes in anemia/non-anemia status together with time-varying covariates […] the model further adjusted only for time-varying **[?]** together with time-varying covariates […] **[?]** the model further adjusted only for time-varying covariates", which seems to suggest that the first model was adjusted for the exposure rather than the exposure being defined as time-varying?

Response

[1.1] Covariates were assessed at each of the 8 follow-ups, while exposure was assessed twice in consenting subjects, as reported under study setting and participants: "Of the initial 1,115 participants, 366 consented to donate a second blood sample" (p. 5, line 126).

We have changed the unclear statement:

"…; it was thus possible to further adjust the multivariable model also for time-varying covariates (…) in subjects with one (prevalent cases) as well as two hemoglobin determinations (incident cases and hemoglobin decline)."

[1.2] Yes, the exposure was "treated as time-varying in the analysis.

There seems to have been an incorrect copy and paste of the text that makes it difficult to interpret the comment. What we wrote was:

"In all subjects with two hemoglobin determinations it was possible to control for both change in the anemia/non-anemia status together with time-varying covariates: the risk associated with baseline anemia resulted moderately increased also with respect to the model further adjusted only for time-varying covariates (Table 5)." In other words: in all subjects with two hemoglobin determinations (last two lines in Table 5) it was possible to control for both time-varying exposure (anemia status) and time-varying covariates and the risk resulted moderately increased (0-11 years: HR, 2.88; 0-7 years: HR, 2.29) also with respect to the model further adjusted "only" for time-varying covariates (first two lines in Table 5; 0-11 years: HR, 1.59; 0-7 years: HR, 1.64). Probably it is that "baseline anemia" (which inaccurately intended to indicate that the follow-up period started from the baseline) that makes the interpretation of what we did unclear.

Changes made to the manuscript:
Under "Statistical analysis": "Moreover, in participants with two hemoglobin determinations it was also possible to investigate how the association between anemia and mortality was affected by time-varying covariates together with time-varying exposure (anemia status)."
Under "Results": "In all participants in the M80+ with two hemoglobin determinations it was possible to control for both time-varying anemia status and time-varying covariates: the risk associated with anemia resulted moderately increased also with respect to the model further adjusted only for time-varying covariates (Table 5)." To this purpose, lines in Table 5 have been re-organized: last line ("Subjects with 2 blood samplings") has been moved below line "Prevalent mild anemia".

2. In the statistical analysis section, it is mentioned that dose response was assessed and that p for trend analyses were carried out. Only one p for trend is in the Results section but none in Table 2 so it is unclear how this analysis was carried out. Further, we can see that some of the estimates and confidence intervals between each dose category are overlapping. The statement "both age groups showed a dose response association" should thus be corrected.

Response

Here there must be some misunderstanding. In response to a Reviewer's comment of the first round of reviews ("The "dose-response relationships" are only stated in the abstract and discussion. This definition of the analysis should be explicitly stated in the methods (…to assess whether a dose-response relationship exist…") and results. Further, it is unclear whether this dose relationship refers to the different cut-off criteria or the comparison of HRs between anemia and mild anemia (which, in the case of anemia, is based on very few individuals with non-mild anemia"), we specified that "we refer to the results reported in Figure 2" and that "we … added the following sentence under the "Statistical analysis": "To assess whether a dose-response relationship existed, hemoglobin concentrations were divided into categories of 1 mg/dL and p for trend analyses carried out.". Thus, the dose relationship analyses have nothing to do with the results reported in Table 2 (which shows the risks associated with the different definitions of anemia and mild anemia). The two *P*s for trend in the young-old and old-old cohorts were already reported in the "Results" section of the original (and revised) manuscript within Figure 2.
We have now added under the "Results" section that:
"Fig. 2 shows the relationship between categorical hemoglobin concentrations and 7-year mortality risk: a dose-response relationship between anemia severity and mortality was observed (*P* for trend <0.0001) and fully-adjusted HRs of death were higher for hemoglobin concentrations below WHO criteria" (for the young-old cohort).
"Relationship between categorical hemoglobin concentrations and 7-year mortality risk in the pooled 80+ cohort was analogous to that in the 65-84 cohort, with higher risk of death for hemoglobin concentrations below WHO criteria and a dose-response relationship between anemia severity and mortality (*P* for trend <0.0001) (Fig 2)." (for the old-old cohort).

3. The study reports the number of individuals who withdrew consent, could not be traced, or refused a second sample. However, it is unclear what were the censoring events during follow-up. Other than death, we would expect the follow-up time to end

when an individual moved, withdrew consent, or reach the end of the study period. Please clarify in the text what were the censoring events.

Response

First it should be taken into account that the only study outcome was death. Thus, in the present study, there are no censoring events other than death: once having accepted to participate (i.e. to donate a second sample), there was no reason/occasion to withdraw consent (the follow-ups of the *H&A* blood study and *M80+* blood sub-study were only "passive" and, in any case, no participant withdrew consent); even if some individuals had moved, their life status would still have been monitored through the Registry Offices of their municipality of residence and/or birth, just as for those who had not changed their residence.

Under the "Study design", we have now added that:

"All participants entered the cohorts on the date of their blood draw and were followed until death or end of the study period. The follow-up period in which death from any cause could be ascertained was 15 years for *H&A65-84* and *M80+* cohorts and 11 years for *H&A85+* cohort. Death was the only censoring event. Dates of death between blood sampling and December 31, 2017 for *M80+* and June 30, 2018 for *H&A*, were obtained from Municipal Registry Offices and Local Health Authorities."

4. **[4.1]** The results were split into two periods (0-7 and 8-15). In my opinion, there is little justification for this split, especially because the mean time between samplings is reported to be 2.2 years (H&A65-84) and 1.7 years (M80+) **[4.2]** For individuals in the second period, was their index date (start of follow-up) the start of the 8th year? We notice in the text that the median follow-up was 7.0 years for the first period and 7.4 years for the second, which suggests that the 8th year is the start of follow-up in the second period. **[4.3]** However, the start of follow-up in the first period was at blood sample. If true, these two different definitions of index date can be problematic.

Response

[4.1] With regard to the "justification of this split", we explained in the original manuscript (under "Statistical analysis") that: "To examine whether the effect of mild anemia was similar over time, two survival analyses were set up: from 0 to 7 years and, in participants who survived the first seven years of follow up, from 8 to 15/11 years". This seemed a reasonable approach to us and we were not the first to split the study period to investigate whether the effect seen along the entire follow-up was similar over time: not to quote ourselves (Riva et al. *Haematologica* 2009), Izaks et al. (*JAMA* 1999) split the 10 years of follow-up of the Leiden study into two periods (0-5 and 5-10 years). Actually, we do not see where the problem is, moreover considering that the results for the entire period of observation were reported and that, following a previous Reviewer's remark, these results were those conveyed in the abstract results.

"… especially because the mean time between samplings is reported to be 2.2 years (H&A65-84) and 1.7 years (M80+)": here as well there is probably some misunderstanding. The "between samplings" refers of course only to the sub-cohorts with two blood samplings. And in these cohorts the risk of mortality associated with the "change in anemia status", "incident anemia" and "[hemoglobin] decline" reported in Tables 4 and 5 was investigated "only" "over 7 years after the second blood sampling". Always in Table 5 in "subjects with two blood samplings" from the *M80+*, the second blood sampling was used only for further adjusting the HR also for time-varying anemia status and thus to test whether the risk of mortality associated with anemia over the

entire period (11 years) or the first seven years was different from the risk further adjusted for only time-varying covariates observed in prevalent cases from the *M80+* (first two lines of Table 5) and from the risk (adjusted neither for time-varying covariates nor for time-varying anemia status) observed in the entire 80-plus cohort (Table 2).
[4.2] Yes, the Reviewer is correct, the index date was the start of the 8th year of follow-up. To avoid any misunderstanding we have now changed in Tables 2 and 5, and in Supplementary Tables S1-S7 the caption of the second period from 8-11/15 to 7-11/15 years.
[4.3] We are not sure we understand where the problem lies. Risks of mortality were estimated for two following periods: 0 to 7 years after the date of blood collection and 7 to 15 (or 11) years after the date of blood collection. All subjects who survived the first seven years of follow-up were included in the analyses of the second period, while all subjects who died along the first seven years of follow-up were of course censored.
We have now added the word "all" before participants (under "Statistical analysis"):
"To examine whether the effect of mild anemia was similar over time, two survival analyses were set up: from 0 to 7 years and, in all participants who survived the first seven years of follow up, from 7 (the start of the 8th year) to 15/11 years".

5. We notice that the median follow-up time for nonanemic patients is 4.1 years and is 7.1 years for anemic patients. However, results showed that mild anemia is associated with an increased risk of mortality. It is probable that the additional 3 years of follow-up for the anemic patients provided an "opportunity" to record more deaths in the anemic group.
Response
Here there may have been a misreading. What we actually wrote was:
"The median follow-up period was 14.0 years with 50,522 person-years of observation and a mortality rate of 4.3 per 100 person-years (4.1 in non-anemic and 7.1 in mild anemic participants)": thus, 4.1 and 7.1 are not the *median years of follow-up*, but the *mortality rates* of non-anemic and anemic participants, respectively.

6. The manuscript would benefit from a clearer organization of the methods and results. The several cohorts, time points, and analyses conducted render the manuscript difficult for the reader to follow as it currently stands.
Response
Assenting to all of the requests the Reviewer made in the first round of revision with regard to the organization of the manuscript, we already rearranged many parts of the Methods and Results. Actually, we do not see what else could be re-organized.
The main cohorts are only *two*: *H&A 65-84* and (*H&A85+* and *M80+*). For some analyses, data were available only for either *H&A85+* or the *M80+* of the pooled (*H&A85+* and *M80+*) cohort. Sensitivity analyses of course had to investigate whether the results would change if different categorizations of the study cohorts (many of which requested by the Reviewers) were considered. It is undeniable that, in the attempt to deal with the limitations of previous studies, the present study tackles the association investigated from different perspectives. In our view however, completeness and accuracy of the results reported should be seen not as barriers but as necessary steps forward in the understanding of the subject at matter. In any case, we think that the

consistency and uniformity of the findings will help the reader to overcome the only seeming complexity of the manuscript.
